# Role of macrophage in ocular neovascularization

**DOI:** 10.1016/j.heliyon.2024.e30840

**Published:** 2024-05-07

**Authors:** Yuanyuan Tu, Yalu Luo, Qingliang Zhao, Yanfeng Zeng, Kai Leng, Manhui Zhu

**Affiliations:** a Department of Ophthalmology, Lixiang Eye Hospital of Soochow University, Suzhou, Jiangsu, China; b Suzhou Medical College, Soochow University, Suzhou, China; c Department of Medical Informatics, Jiangsu Province Hospital, The First Affiliated Hospital of Nanjing Medical University, Nanjing, 210029, China

**Keywords:** Macrophage, Neovascularization, Corneal, Retinal, Choroidal, VEGF

## Abstract

Ocular neovascularization is the leading cause of blindness in clinical settings. Pathological angiogenesis of the eye can be divided into corneal neovascularization (CoNV), retinal neovascularization (RNV, including diabetic retinopathy and retinopathy of prematurity), and choroidal neovascularization (CNV) based on the anatomical location of abnormal neovascularization. Although anti-Vascular endothelial growth factor (VEGF) agents have wide-ranging clinical applications and are an effective treatment for neovascular eye disease, many deficiencies in this treatment strategy remain. Recently, emerging evidence has demonstrated that macrophages are vital during the process of physiological and pathological angiogenesis. Monocyte-macrophage lineage is diverse and plastic, they can shift between different activation modes and have different functions. Due to the obvious regulatory effect of macrophages on inflammation and angiogenesis, macrophages have been increasingly studied in the field of ophthalmology. Here, we detail how macrophage activated and the role of different subtypes of macrophages in the pathogenesis of ocular neovascularization. The complexity of macrophages has recently taken center stage owing to their subset diversity and tightly regulated molecular and metabolic phenotypes. In this review, we reveal the functional and phenotypic characterization of macrophage subsets associated with ocular neovascularization, more in-depth research is needed to explore the specific mechanisms by which macrophages regulate angiogenesis as well as macrophage polarization. Targeted regulation of macrophage differentiation based on their phenotype and function could be an effective approach to treat and manage ocular neovascularization in the future.

## 1 Background

Ocular angiogenesis is the formation of new vessels from the existing blood vessels in the eyes and is the major cause of blindness in clinical settings [1]. Angiogenesis occurs in physiological and pathological environments, playing a causal role in various diseases [2]. Once a functional adult vascular system is fully formed, blood vessels become quiescent. However, the growth potential of small blood vessels is preserved and plays a role in wound healing and tissue regeneration. Under pathological conditions, including hypoxia, inflammation, and tumorigenesis, diseased cells produce abnormal amounts of angiogenic factors, thereby inducing the formation of excessive neovascularization [3].

**Fig. 1:**
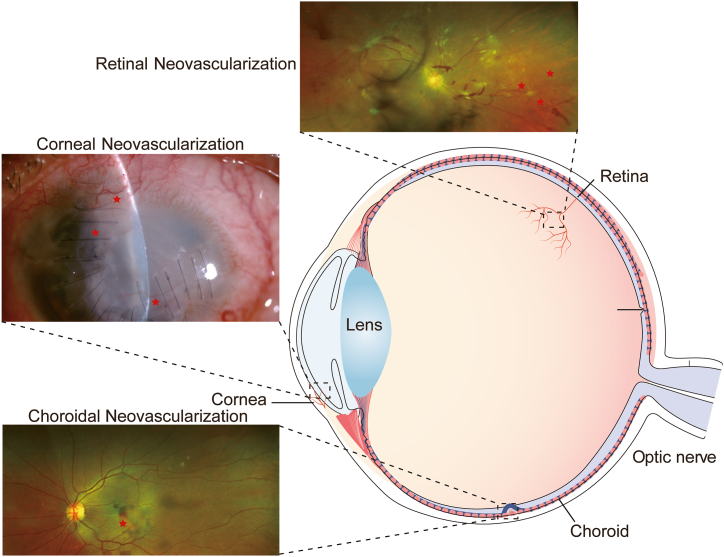
Pathological angiogenesis of the eye can be divided into corneal neovascularization, retinal neovascularization, and choroidal neovascularization based on the anatomical location of abnormal neovascularization. Neovascularization in the clinical examination results are marked with “☆“.

Based on the anatomical location of abnormal neovascularization, pathological neovascularization of the eye can be divided into corneal neovascularization (CoNV), retinal neovascularization (RNV), and choroidal neovascularization (CNV) (Fig. 1). The invasion of neovascularization leads to corneal stroma opacity and irregularity of the ocular surface. Intraocular neovascularization leads to retinal vascular leakage, retinal edema, and hemorrhage [3,4]. Vascular endothelial growth factor (VEGF) primarily leads to ocular neovascularization. VEGF promotes the proliferation and migration of endothelial cells and results in the formation of capillary-like tubular structures [5]. At present, ophthalmologists use anti-VEGF drugs commonly apart from traditional treatments, such as photocoagulation and photodynamic therapy (PDT). The development of anti-VEGF drugs has gone through three stages, from the initial base RNA (such as Macugen) to the monoclonal fragment (such as Bevacizumab), and presently, the fusion protein (such as aflibercept) [6]. Although anti-VEGF agents are widely used in clinical settings and are an effective treatment for neovascular eye diseases, several deficiencies in this treatment strategy remain [4,7]. Anti-VEGF agents are expensive, and patients often require multiple injections, imposing a great economic burden on these patients. Although only a small amount of the drug is injected into the eye, the risk of arterial hypertension and embolism remains [8]. Studies have shown that anti-VEGF treatment can potentially increase the progression of geographic atrophy [9].

**Fig. 2:**
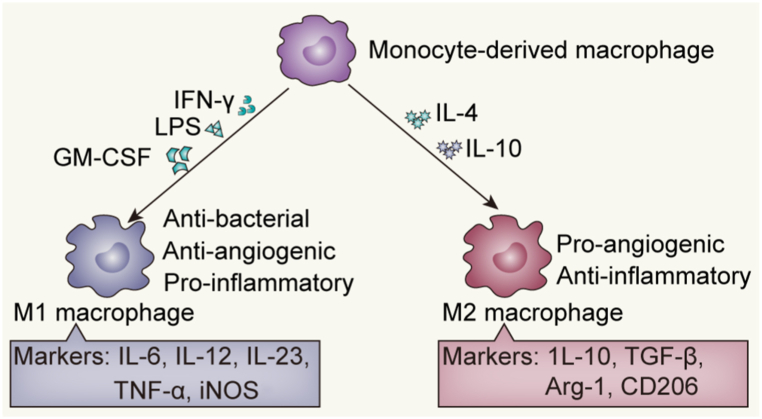
Macrophages are classically activated by IFN-γ, LPS, or GM-CSF stimulation and produce high levels of IL-12, IL-23, TNF-α, IL-6 and iNOS (M1 macrophage). M1 macrophages have important anti-bacterial and pro-inflammatory properties and exert anti-angiogenic effects. During alternative activation, macrophages polarize into a pro-angiogenic phenotype in the presence of IL-10 or IL-4, a phenomenon characterized by high levels of IL-10, TGF-β, CD206, and Arg1 expression (M2 macrophages).

Several recent studies have demonstrated that macrophages are critical in angiogenesis [10,11]. Monocyte-macrophage lineage is diverse and plastic and can shift between different activation modes to exert different effects. Unique stimuli activate macrophages into subtypes with distinct molecular phenotypes and functions [[12], [13], [14]]. Macrophages are classically activated by stimulation with interferon-γ (IFN-γ), lipopolysaccharide (LPS), or granulocyte macrophage-colony stimulating factor (GM-CSF), and secrete high levels of interleukin-12 (IL-12), IL-23, tumor necrosis factor-α (TNF-α), IL-6, inducible nitric oxide synthase (iNOS) and low levels of IL-10, transforming growth factor β (TGF-β), and arginase 1 (Arg1) (M1 macrophage). M1 macrophages have important anti-bacterial and pro-inflammatory properties and exert anti-angiogenic effects. During alternative activation, macrophages polarize into a pro-angiogenic phenotype in the presence of IL-10 or IL-4, a phenomenon characterized by high levels of IL-10, TGF-β, CD206, and Arg1 expression and low levels of M1 cytokines (M2 macrophages) (Fig. 2) [15]. Due to the obvious regulatory effect of macrophages on inflammation and angiogenesis [2,16], these are increasingly being studied in the field of ophthalmology. Herein, we review the effect and mechanism of macrophages in ocular angiogenesis and summarize the results from angiogenesis-related studies.

## 2 Main text

### 2.1 Macrophages in CoNV

Corneal avascularity is critical for maintaining corneal transparency and is the basis for good vision. The cornea contains anti-angiogenic factors that sustain avascular properties known as “angiogenic privilege” [17]. However, when the balance between pro-angiogenic and anti-angiogenic molecules is disrupted, the “privileged angiogenesis” of the cornea is lost. Many diseases, including alkali burns, inflammatory disorders, stromal ulceration, and infectious keratitis induce CoNV [18,19]. Corneal transparency reduces if the blood vessels grow into the optical zone or immature capillaries experience side effects, such as bleeding and lipid leakage. Macrophages are present in the immune-privileged cornea, higher towards the periphery of the corneal stroma [20], and are pivotal in CoNV.

In a previous study, a single-cell suspension from the synovial fluid of patients with rheumatoid arthritis was injected into the corneal stroma at 1–1.5 mm from the corneal limbus. The group with the highest macrophage content consistently showed neovascularization induction in the rat corneas [21]. Macrophages activated by mineral oil lumbar injection were transplanted into corneal micro pockets. Activated macrophages significantly induced CoNV, which peaked at day 5, while the normal corneas and the negative control group showed less CoNV [22]. Macrophage depletion significantly decreased the formation of CoNV [23]. Liu et al. [24] established a mouse model of CoNV induced by alkali burn. Their results demonstrated that IL-17A levels were elevated after alkali injury and that recombinant IL-17A (m IL-17A) administration exerted significant pro-angiogenic effects. They examined the normal corneas and found no infiltration of macrophages, and the alkali-injured corneas had approximately 30.5 ± 2.4 % M1 macrophages and 4.5 ± 0.5 % M2 macrophages. mIL-17A treatment increased the percentage of M1 macrophages to nearly 41.5 ± 3.2 %, whereas the percentage of M1 macrophages decreased to 22.7 ± 3.1 % after treatment with an anti-mouse IL-17A antibody [24]. In vitro treatment of macrophages with IL-17A induced increased secretion of potent angiogenic molecules, VEGF and IL-6 [24]. These findings suggested that alkali burn promoted the infiltration of M1 macrophages in the corneas, and IL-17A stimulation further increased the infiltration levels. Additionally, IL-17A facilitated the secretion of angiogenic molecules from macrophages including VEGF and IL-6, subsequently enhancing the formation of CoNV [24]. Hos, Deniz et al. [25] demonstrated the effect of IL-10-activated macrophages in inflammatory CoNV. IL-10 expression was not verified but was dramatically increased in macrophage-infiltrating inflamed cornea after corneal injury. Macrophages stimulated in vitro with IL-10 showed an upregulation of pro-lymphangiogenic VEGF-C. Consistently, in IL-10 deficient mice, corneal inflammation led to decreased VEGF-C and corneal lymphangiogenesis. Whereas, the deletion of IL-10 did not affect corneal angiogenesis. These findings indicated that in the inflammatory response of cornea, IL-10 stimulation induced an anti-inflammatory but pro-lymphangiogenic VEGF-C-secreting macrophage phenotype. Macrophages promoted lymphatic vessels' activation and growth as well as the faster resolution of inflammation but the macrophage phenotype did not affect corneal angiogenesis [25]. The role played by macrophages in CoNV is related to their stimulating factors and the activating subtypes.

Although numerous studies have reported that macrophages are pro-angiogenic, Lu et al. revealed that macrophages can also exert the antiangiogenic effects [26]. The infiltration of CX3CR1-positive macrophages intraocularly attenuated alkali-induced CoNV, which was mediated by producing antiangiogenic factors such as thrombospondin-1(TSP-1) and a disintegrin and metalloproteinase with thrombospondin repeats-1 (ADAMTS-1) [26,27]. They also found that macrophage depletion has no apparent effects on alkali-induced CoNV, which is in contrast to other studies [23]. This may be due to the opposite effect of CCR2-expressing and CX3CR1-expressing macrophages on angiogenesis. CCR2-deficient mice showed reduced CoNV while CoNV increased in CX3CR1-deficient mice [27].

Functioning as the major source of angiogenic factors is one of the main mechanisms by which macrophages promote angiogenesis [28,29]. Macrophage-secreted cytokines such as VEGF and TNF-α promote the proliferation, migration, and tube formation of endothelial cells [30]. VEGF is the main factor in angiogenesis and has a critical role in the progression of CoNV [31]. In addition, anti-VEGF agents are effective in the treatment of CoNV [32,33].

Local or subconjunctival injection of ranibizumab, bevacizumab, and aflibercept significantly reduce CoNV [32,34]. In addition to these well-known therapeutic measures, numerous agents or targets are effective in the inhibition of CoNV by modulating macrophages. Li et al. found that celastrol-loaded nanomicelles (CNMs) suppressed CoNV induced by macrophage activation in a model of rat corneal pocket [35]. CNM administration suppressed the expression of VEGF and matrix metalloproteinase-9 (MMP-9) in the activated macrophages and the cornea. These effects might be mediated through the hypoxia-inducible factor-1 alpha, mitogen-activated protein kinase (MAPK), and nuclear factor kappa-light-chain-enhancer of activated B cells (NF-κB) signaling pathways in macrophages [35]. Glutaminase 1 inhibitor markedly decreased the infiltration of polarized M2 macrophages and suppressed the progression of CoNV both in vivo and in vitro. CB-839 (glutaminase 1 inhibitor) treatment attenuated the secretion of proangiogenic factors including VEGF and platelet-derived growth factor-BB in M2 macrophages [36]. Other molecules, such as stromal-derived factor-1 (SDF-1) α, TNF-α, and chemokine C–C motif chemokine ligand 3 (CCL3), enhanced angiogenesis in alkali-induced CoNV by promoting macrophage infiltration and VEGF secretion [[37], [38], [39]]. However, the above studies have not analyzed the phenotypes of the macrophages. The inhibitors targeting these molecules above may be useful for the treatment of CoNV and merit further investigations. MicroRNAs (miRNAs) are highly conserved small non-coding RNAs that are involved in regulate CoNV [40]. Wang et al. reported that miR-497 overexpression inhibited the maturation, polarization, and recruitment of macrophages by restraining the signal transducer and activator of transcription 3 (STAT3) pathway. miR-497 protects the corneas, prevents alkali burn injury and inhibits neovascularization through a finely regulated network comprising the miR-497/IL-6/STAT3/VEGFA axis [40]. Taken together, strategies targeted at the regulation of macrophages are effective in the treatment of CoNV. However, according to the existing data, no speculation or conclusion can be drawn on whether targeting different macrophage populations or different activation phenotypes regulates CoNV.

## 3 Macrophages in RNV

### 3.1 Proliferation diabetic retinopathy (PDR)

Diabetic retinopathy (DR) is the main cause of irreversible vision impairment in working-aged people [41,42]. Proliferation diabetic retinopathy (PDR) is characterized by RNV with hyperproliferation being the most refractory form of the disease in its advanced stages. Vitreous hemorrhage and/or traction retinal detachment is the leading cause of vision loss in PDR. Macrophages are predominantly found in the proliferative membranes of patients with PDR. Furthermore, macrophages are involved in the proliferative membranes of PDR patients with or without vitreous hemorrhage [43]. The number of macrophages increases in fibrovascular membranes, vitreous samples, and iris of PDR patients compared with nondiabetic controls [[44], [45], [46], [47]]. In the iris, macrophage staining is more pronounced in diabetics than in non-diabetics. The staining of macrophages is prominent in the neovascular membranes compared to the iris of diabetic patients [47]. Macrophages polarized towards M2 phenotypes exhibit a higher potential for angiogenesis in the pathogenesis of PDR. Wang et al. found that M2 macrophages were significantly increased in both PDR and diabetic macular edema (DME) patients compared to the healthy controls. Welch's *t*-test showed that only M2 macrophages were markedly elevated in the macula of patients [48]. Combined analysis of differential expression and co-expression revealed the presence of M2 macrophage-associated hub genes in PDR. Of these, collagen type V alpha 2, caldesmon 1, collagen type VI alpha 3, coronin-like actin-binding protein 1C, and calumenin were increased in fibrovascular membranes and may be the potential biomarkers for PDR [44]. As a specific biomarker for M2 macrophage activation, soluble cluster of differentiation 163 (sCD163) is up-regulated in the vitreous of PDR patients, and sCD163 showed a significant positive correlation with VEGF [46]. The density and number of microvessels in the preretinal membrane of PDR patients are also positively correlated with stromal cells expressing CD63 [46].

Taken together, these findings suggest that macrophages, especially M2 macrophages, appear to play a more important role in the pathogenesis of PDR. More studies need to explore the relationship between PDR and M2 macrophages to translate these findings into meaningful therapeutic clinical treatments targeting M2 macrophages.

### 3.2 Oxygen-induced retinopathy (OIR)

Retinopathy of prematurity (ROP) is the main cause of blindness in infants due to exposure to elevated oxygen levels [49]. Oxygen-induced retinopathy (OIR) is a widely used neonatal animal model to reproduce the proliferative phase of ROP. RNV and detachment characterize severe ROP. In recent decades, macrophages have been reported to play an important role in RNV, specifically in ROP [48,50]. The number of macrophages increases significantly in the eyes of the oxygen-treated mice compared to normal controls [51]. After four times depletion of macrophages by intraperitoneal injection of clodronate-liposomes, retinal avascular area and the neovascular area reduce drastically [52]. Vitreous macrophages participate actively in pathologic angiogenesis triggered by retinal ischemia. Intravitreal injection of peritoneal macrophages revealed only occasional migration of the macrophages into retinas of wild-type mice(P8), whereas transplantation is more vigorous in OIR mice, usually around neovascularization (P17) [53]. Microglial cells/macrophages localize in the area of neovascularization in oxygen-treated mice at P17 [54]. These research findings provide a theoretical basis for the exploitation of antiangiogenic treatments targeting ocular macrophages.

Several studies demonstrated that M1/M2 phenotype of macrophages in the retina of mice is altered after successful modeling of OIR [55,56]. The number of M1 macrophages increases at P13 and peaks at P17; it returns to a relatively normal level at P21. The number of M2 macrophages in mice with OIR rapidly increase from P13 to P21. The decrease in macrophage M1/M2 ratio implicates a shift in macrophage polarization towards the M2 subtype. In early OIR, M1 macrophages were shown to interact with endothelial tip cells in the anterior part of the vasculature of both the central and peripheral retina, while M2 macrophages embrace the emerging vessel and buddingly bridge with adjacent vessels, suggesting a function in promoting tip cell fusion in the later stages [56]. During the polarization of M1 microglial/macrophages, the NF-κB-STAT3 axis is activated, along with an enhanced secretion of inflammatory cytokines, including TNF-α, IL-6, and IL-1β. Signaling pathway IL-4-STAT6-PPAR-γ is activated during M2 microglial/macrophages polarization, which leads to suppression of inflammatory cytokines and spontaneous regression of the neovascular cluster [55]. Therefore, modulating the conversion of macrophages towards the M2 phenotype is a potentially clinically valuable approach for the treatment of ROP.

M2 macrophages are the main immune cells infiltrating in the retinas of OIR rats [48]. Zhou et al. [57] also demonstrated that it is M2 macrophages but not M1 macrophages, that are highly expressed in the retinas of OIR mice. Selective inhibition of M2 macrophages decreases the size of neovascular tufts and the avascular area. Conversely, intravitreally injection of bone-derived M2 macrophages increases pathological neovascular tufts and avascular area compared with the normal controls [57].

Bone-derived M2 macrophages were cocultured with human retinal endothelial cells (HRECs) and the results showed that M2-polarized macrophages promote the tube formation ability of HRECs in an in vitro experiment [57]. Therefore, M2 macrophages appear to play a more important role than M1 macrophages in promoting pathological RNV in the OIR model.

NF-κB is a multidirectional, multifunctional nuclear transcription factor that is implicated in RNV [58]. RNV reduces significantly after treatment with the NF-κB inhibitor, pyrrolidinedithiocarbamate (PDTC) [59,60]. Moreover, PDTC decreases the levels of M1-polarized macrophages as well as M1-macrophage-associated cytokines. In contrast, the abundance of M2-polarized macrophages and their associated cytokines increases substantially. Blocking NF-κB signaling inhibits RNV in mice with OIR through promoting M1 macrophage polarization towards M2 type. Furthermore, NF-κB mediates macrophage polarization by regulation of MAPK activation and Notch1 inhibition [60]. In addition to PDTC, pigment epithelium-derived factor (PEDF) exerts similar effects. PEDF decreases neovascularization and macrophage recruitment. Flow cytometry analysis showed that M1 and M2 subtype polarization reduced after PEDF treatment in the retinas of mice. PEDF dampened the RNV by mediating the polarization of macrophages in OIR [61]. NF-κB and PEDF play a key role in regulating RNV by mediating macrophage polarization in mice with OIR; NF-κB and PEDF may serve as therapeutic targets. More studies are required to explore the macrophage polarization mechanism and identify more effective targets.

In summary, monocytes are recruited to the vitreous membrane and retina under oxygen-deprived conditions. NF-κB, PEDF, and other molecules polarize and activate the monocytes to M1 and M2 macrophage subtypes. M2 macrophages embrace the emerging vessel, form a budding bridge with adjacent vessels, and promote tip cell fusion in the later stages, thereby promoting RNV in OIR.

## 4 Macrophages in CNV

Age-related macular degeneration (AMD) is the main cause of blindness in developed countries [62]. Wet form of AMD, or vascular AMD leads to vision loss due to CNV. Immune activation plays a critical role in the pathogenesis of CNV and numerous studies have reported the presence of macrophages within CNV [63]. Macrophages in tissues are mainly derived from monocytes in peripheral blood. Recruitment of blood-derived macrophages appears to correlate more strongly with CNV formation than recruitment of resident microglia [64]. One day after laser photocoagulation of the mouse retina, macrophages invaded the sites of laser injury and peaked at day 3^65^. Treatment of mice with CL2MDP-containing liposomes (CL2MDP-lip) sufficiently depleted of circulating monocytes and choroidal macrophages. Macrophage-depleted mice showed a smaller area of CNV as well as less vascularity and cellularity of CNV lesions compared to the controls [65]. Moreover, the administration of CL2MDP-lip 2 days before laser injury was more beneficial than immediately after. CNV volume was correlated with the protein level of VEGF and the number of infiltrating macrophages [66]. These reports confirm that macrophages exert promotive effects during the formation of CNV. However, Apte et al. [67] found that the area of CNV lesions decreased with the recruitment of macrophages. Macrophages were prevented from entering the eye to promote CNV formation. Simultaneously, the area of CNV was reduced by direct injection of macrophages into the vitreous cavity of the mice, indicating that macrophages inhibit CNV formation in IL-10^−/−^ mice [67]. IL-10 is an important stimulator of macrophage M2-type polarization, therefore, the knockdown of IL-10 may increase the M1/M2 ratio of macrophages, leading to inhibition of neovascularization [67].

Macrophages with one phenotype can easily be converted to another as the microenvironment changes [68]. An increased level of M2 CCL22 (C–C motif chemokine 22), decreased level of M1 CXCL11(C-X-C motif chemokine 11), and decreased M1/M2 ratio (CXCL11/CCL22) have been observed in the macular choroidal trephines (MCT) of old compared to young non-AMD. Compared to non-AMD MCT, AMD MCT has higher CXCL11 levels, similar CCL22 levels, and an increased M1/M2 ratio [69]. The expressions of M1-related markers (including iNOS, TNF-α, IL-6, CXCL10, and CXCL11) are significantly upregulated in the early phase of laser-induced CNV model in mice, while M2-related markers (including CD163, CD206, and CCL22) were slightly upregulated in intermediate phase and continued in the late phase. Additionally, a higher ratio of M2/M1 was found in the mouse model as well as the aqueous humor of patients with neovascular AMD (nAMD) [70,71]. The injection of M2 macrophages into the vitreous cavity of mice exacerbates the formation of CNV lesions, while M1 macrophages ameliorate them [71]. Therefore, it is possible that M2 macrophages may play a more significant role during the progression of CNV.

The factors affecting macrophage polarization are clearly multifactorial. Aging is an important factor in the polarization of macrophages. Macrophages are predominantly of the M2-type in the eyes of patients with AMD and normal aged eyes, whereas in young eyes, these are predominantly M1^69^. Zhao et al. [72] also demonstrated that M2 macrophages are pronounced in the eyes of aged mice. In the laser-induced mouse CNV model, aged mice had larger CNV volumes than younger mice, and aged mice showed more aggregates of M2 macrophages at the CNV lesions. The macrophages derived from aged or young mice were injected into the eyes of aged mice respectively the day of laser treatment; CNV lesion was detected on day 7 after laser photocoagulation. The macrophages obtained from young mice markedly suppressed CNV formation, while those from aged mice showed no such inhibitory effects [73]. Fas ligand (FasL) serves a nexus role in aging and macrophage polarization [72]. The level of FasL was up-regulated in the eyes of mice with age, and blood levels of soluble FasL (sFasL) were higher in aged mice than in young mice. sFasL promoted cytokine production in aged macrophages as well as the M2 macrophages. Compared with M1 macrophages in young mice, senescent M2 macrophages showed elevated Fas expression and exhibited increased migration toward CNV lesions in response to sFasL [72]. Aging increased Rho-associated coiled-coil-containing protein kinase 2 (ROCK2) signaling, promoting the expression of proangiogenic M2-like macular-degeneration-associated macrophages. Selective inhibition of ROCK2 increased the M1/M2 ratio, decreased CNV formation, and restored the physiological macrophage balance found in young ones [71].

Aberrant expression of non-coding RNAs is another important factor in the regulation of macrophage polarization [16]. MicroRNA-155 (miR-155) is down-regulated in PGE2-treated marrow-derived macrophages and laser-induced CNV mice. miR-155 mimics transfection and decreases the level of M2 markers including Arg-1, YM-1 by targeting C/EBPβ in in vitro and in vivo experiments [74]. miR-505 expression was elevated in the RPE/choroid complexes of patients with AMD and laser-induced CNV mouse model. Inhibition of miR-505 significantly suppressed the expression of M2-specific markers Arg-1 and YM-1, while showing no effect on M1-specific markers TNF-α and iNOS. At the molecular level, miR-505 promoted macrophages polarize toward M2 type by directly targeting and negatively regulating TMEM229B [75]. In addition to miRNAs, long non-coding RNA (lncRNAs) are involved in macrophage polarization in CNV. LncRNA NEAT1 is markedly up-regulated in the CNV mouse model, as evidenced in profiling microarray. Knockdown of lncRNA NEAT1 decreased Arg-1 and YM-1, whereas TNF-α and iNOS genes elevated significantly in the CNV mouse model. LncRNA NEAT1 acts as miR-148a-3p sponge to regulate PTEN expression and macrophage M1/M2 polarization in CNV formation [76]. Thus, the regulation of macrophage polarization and CNV progression by lncRNAs are closely related, providing strong evidence for modulating macrophage polarization status for the therapy of CNV.

Colony-stimulating factor 1 (CSF1) released from HCVECs under conditions of hypoxia promotes macrophage M2 polarization in CNV models. CSF1, also known as macrophage-CSF, regulates macrophage recruitment and polarization [77]. CSF1 binds to the CSF1 receptor (CSF1R) and promotes the migration and M2 polarization of macrophages by activating the phosphoinositide 3-kinase (PI3K)/protein kinase B (AKT)/forkhead box protein O1(FOXO1) axis. CSF1-neutralizing antibodies increase the expression of the M1-type macrophage marker but decreased the expression of the M2-type marker. Moreover, CSF1/CSF1R also ameliorates laser-induced CNV formation in mice [78]. CSF1 released from HCVECs under hypoxic conditions is important for inducing macrophage M2 polarization and CNV formation.

In addition to the first-line anti-VEGF therapy [79], many agents or targets that regulate macrophage polarization play a critical role in the treatment of neovascular AMD (Table 1). Melatonin was demonstrated to attenuate CNV and decreased vascular leakage and proliferation by regulating the polarization of macrophage/microphage from M2-type to M1-type, mediated by inhibiting the RhoA/ROCK signaling pathway [80]. Triptolide, a chemical exact isolated from the Chinese herb Tripterygium wilfordii Hook. F, is a potential angiogenesis inhibitor [81]. Triptolide reduces the areas of CNV in a dose-dependent manner. Infiltrated M2 macrophages and the ratio of M2/F4/80^+^ macrophages are downregulated by using Triptolide significantly, consistent with the decreased protein levels of VEGF, intercellular adhesion molecule −1, TNF-α, and IL-6 [82]. Introvitreal injection of Triptolide with the novel sustained-release nano-drug delivery system (TP-nanolip-PEG or TP-nanolip-APRPG) suppresses the polarization of M2 macrophages and enhances inhibition of Triptolide on laser-induced CNV and subretinal fibrosis [83,84]. Doxycycline inhibits M2-polarization of both human and mouse marrow-derived macrophages in a dose-dependent manner [85]. It decreases laser-induced CNV formation in mouse or rat models and enhances the effects of current antiangiogenic therapies in CNV that are promoted by the M2-macrophages [85,86]. The agents are all demonstrated to be efficacious in the treatment of CNV, mostly by regulating the phenotype of macrophages.

**Table 1:** Agents or targets that regulate macrophage polarization in CNV.

Drugs	Targets	Research objects	Macrophage polarization	Role in CNV	References
Rho-associated kinase (ROCK)-2 siRNA	ROCK-2	Human CNV membranes, Monkeys, Mice, U937 cells and RAW264.7 cells	M1↑, M2↓	Inhibit	[71]
miR-155 mimic	miR-155/CCAAT/enhancer binding protein (C/EBP) β	Mice and BMDMs isolated from mice	M2↓	Inhibit	[87]
miR-505 inhibitor	miR-505/Transmembrane Protein 229B (TMEM229B)	Human retinal tissues, Mice and BMDMs isolated from mice	M2↓	Inhibit	[75]
LncRNA NEAT1 smart silencer	NEAT1/miR-148a-3p/Phosphatase and Tensin Homolog deleted on Chromosome 10 (PTEN)	Mice and BMDMs isolated from mice	M2↓	Inhibit	[76]
CSF1 Neutralize Antibody	CSF1/CSF1R/PI3K/AKT/FOXO1 axis	Mice, Human choroidal vascular endothelial cells (HCVECs) and Human peripheral BMDMs	M1↑, M2↓	Inhibit	[78]
Melatonin	RhoA/ROCK	Mice	M2→M1	Inhibit	[80]
Triptolide	/	Mice, THP-1 human monocyte cells and human umbilical vein endothelial cells (HUVECs)	M2↓	Inhibit	[[82], [83], [84]]
Doxycycline	/	Mice, THP-1 cells and BMDMs isolated from mice	M2↓	Inhibit	[85,88]
SiR- tissue inhibitor of metalloproteinases–3 (TIMP-3)	TIMP-3	Mice and BMDMs isolated from mice	M2↑	Promote	[89]
Rapamycin	mTORC1	Mice, THP-1 cells and ARPE-19 cells	M1↓, M2↑	Promote	[90]
Chitinase-3-Like-1 (CHI3L1) Neutralize Antibody	MAPK	nAMD Patients, mice, RF/6A cells and RAW264.7 cells	M2↑	Promote	[91]
IL-17A Neutralize Antibody	IL-17A	Mice, HUVECs and RAW264.7 cells	M1↓, M2↑	Promote	[92]

The targets regulating macrophage polarization seem multifactorial (Table 1). Tissue inhibitor of metalloproteinases–3 (TIMP-3) is critical in the polarization of macrophages in several tissues, including the choroid [[89], [93], [94]]. Knockdown of TIMP-3 stimulates M2 polarization in BMDMs and laser-induced mice models. Moreover, the lack of TIMP-3 accelerated CNV formation suggests that TIMP-3 inhibition is associated with pro-angiogenic M2 macrophages [89]. Leukotriene B4 receptor 1 (BLT1) is upregulated in M2 macrophages. After laser treatment, the BLT1-positive M2 macrophages were upregulated in the eyes of aged mice. BLT1 deficiency attenuated VEGFA production in M2 macrophages and augmented CNV formation [95]. Inhibiting the activity of the mechanistic target of rapamycin complex 1 (mTORC1) shifted macrophage polarization toward the M2-type and was reversed by mTORC1 hyperactivation. mTORC1 activity in macrophages regulates the expression of cytokines closely related to CNV development [90].

Overall, interventions using the drugs and targets mentioned above critically regulate CNV (Table 1). Targeted regulation of macrophage differentiation according to their phenotype and function may be effective in treating and managing CNV in the future. However, none of the above studies reported the specific mechanisms of macrophage polarization and the regulation of cytokines following polarization.

## 5 Conclusions

**Fig. 3:**
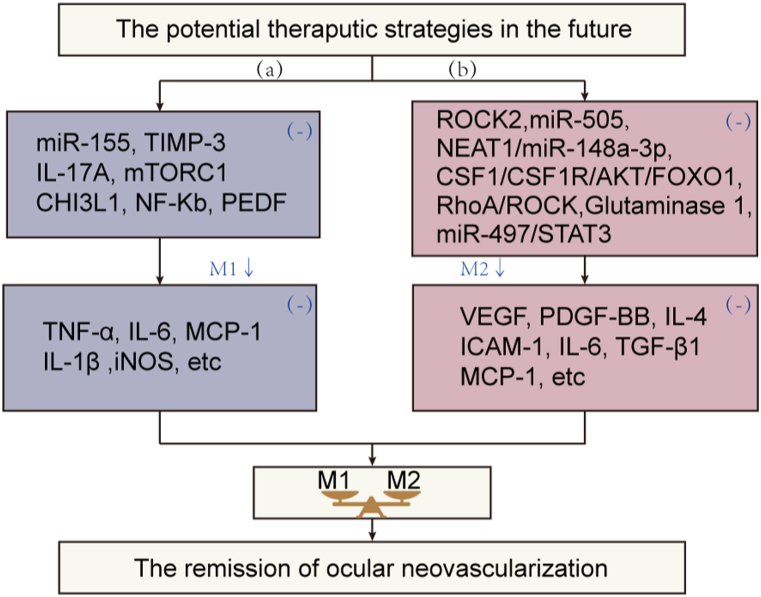
Future directions of treatment for macrophage polarization-mediated ocular neovascularization. (a). Future treatment directions in M1 polarization-mediated ocular neovascularization. (b). Future treatment directions in M2 polarization-mediated ocular neovascularization.

Ocular neovascularization is the leading cause of blindness in a variety of ocular diseases, characterized by forming of new vessels from the existing blood vessels in the eye. Pathological angiogenesis of the eye can be divided into CoNV, RNV, and CNV according to the anatomical location of abnormal neovascularization. Macrophages play a critical role in angiogenesis, both physiological and pathological, and are increasingly being studied in the field of ophthalmology. This article reviews the role and mechanism of macrophages in ocular neovascularization and summarize the results from neovascularization-related studies. According to the different phenotype macrophages and their respective roles in the CoNV, RNV and CNV, some critical targets are proposed and summarized for the treatment of ocular neovascularization via regulating macrophage polarization (Fig. 3). Nevertheless, more in-depth research is needed to explore the specific mechanisms by which macrophages regulate angiogenesis as well as macrophage polarization. Targeted regulation of macrophage differentiation based on their phenotype and function could be an effective approach to treat and manage ocular neovascularization in the future.

## Data availability statement

Data will be made available on request.

## CRediT authorship contribution statement

**Yuanyuan Tu:** Writing – original draft, Funding acquisition, Conceptualization. **Yalu Luo:** Visualization, Software, Resources, Conceptualization. **Qingliang Zhao:** Visualization, Software, Resources, Conceptualization. **Yanfeng Zeng:** Visualization, Software, Resources, Investigation. **Kai Leng:** Software, Resources, Formal analysis. **Manhui Zhu:** Writing – review & editing, Conceptualization.

## Declaration of competing interest

The authors declare that they have no known competing financial interests or personal relationships that could have appeared to influence the work reported in this paper.
